# Do Athletes Cook? A Systematic Scoping Review of Culinary Nutrition in Athletes

**DOI:** 10.1111/nbu.70012

**Published:** 2025-05-07

**Authors:** Rachael Camp, Oliver C. Witard, Michèle Renard, Ciarán Ó. Catháin, Fiona Lavelle

**Affiliations:** ^1^ Department of Nutritional Sciences School of Life Course & Population Sciences, Kings College London London UK; ^2^ Centre for Human & Applied Physiological Sciences King's College London London UK; ^3^ Department of Sport and Health Sciences Technological University of the Shannon Westmeath Ireland; ^4^ SHE Research Centre Technological University of the Shannon Westmeath Ireland

**Keywords:** cooking, dietary intake, nutrition knowledge, performance, sports

## Abstract

Applied sports nutrition is fundamental to athlete health, performance and training adaptation; hence, culinary skills are paramount to meet physiological demands. With the decline in domestic cooking, culinary nutrition has emerged as a priority for research and education. However, limited information exists regarding culinary research in athletes. The aim of this scoping review was to collate, map and overview the current body of literature regarding culinary nutrition in athletes while also identifying existing knowledge gaps. The PRISMA‐ScR guidelines were used. Five electronic databases were searched in March 2023. Data extraction was conducted using a custom form. A critical appraisal was undertaken, data were charted in narrative and numerical format, and thematic analysis was conducted mapping relationships between studies and identifying knowledge gaps. Seven primary research studies were included, two qualitative and five quantitative study designs, with only one randomised controlled trial. Four studies were conducted in North America and four studies included athletes from multiple sports. Delivery, content and duration of interventions varied, with limited detail provided in most studies. All intervention studies included a practical culinary element. The use of validated measurement tools was limited. One study received a positive rating in the critical appraisal. Culinary nutrition research in athletes is grossly limited and warrants further investigation. Specifically, a focus on methodological issues is required to improve the quality of evidence, with the inclusion of well‐designed studies, use of validated measurement tools, implementation of clear theoretical frameworks and clear intervention descriptions to assist in strengthening the culinary nutrition research field.

## 1 Introduction

### 1.1 Cooking as a Mechanism for Improving Diet Quality and Health Outcomes

Noncommunicable diseases (NCDs), including cardiovascular diseases, cancers and diabetes, are responsible for 74% of all deaths worldwide, equating to 41 million individuals annually (World Health Organization, 2023). A key modifiable risk factor for NCDs is unhealthy diets, with improvements in diet quality having the potential to prevent > 11 million deaths annually (Wang et al. 2019). Cooking has received recent attention as a potential solution to poor dietary intake in the general population, as cooking skills and consumption of home cooked meals have been associated with improvements in diet quality and dietary intake, including a decreased intake of total energy, fat and sugar and an increased consumption of fruit and vegetables (Wolfson and Bleich 2015; Mills et al. 2017a; McGowan et al. 2016; Lavelle et al. 2020). Furthermore, cooking has been associated with other health markers and outcomes such as lower adiposity, normal BMI, normal HbA1c and low cholesterol, all clinically significant indicators of reduced cardiometabolic and type 2 diabetes risk (Wolfson and Bleich 2015; Mills et al. 2017a; Lavelle et al. 2020). This observation highlights the utility of cooking as a practical tool to improve overall diet quality by tailoring the dietary intake of specific populations, including athletes, depending on their requirements.

With the decline in domestic cooking (Griffith et al. 2022), culinary nutrition, also known as culinary medicine, has emerged as a research and education area for teaching cooking and food skills. The discipline teaches the science, theory and practicalities of combining food groups to create adequate nutrient‐dense meals that can be tailored to the individual's nutrition requirements (Marcus 2013). A recent systematic scoping review by Asher and colleagues synthesised current research describing culinary nutrition education programmes in individuals working or training in health, adult education or culinary roles (Asher et al. 2022b). Thirty‐three studies were identified that delivered culinary interventions to general population groups, health professionals or students. Findings showed changes in nutrition knowledge, dietary intake and behaviour, highlighting the relevant benefits that could be translated to other populations. However, with culinary nutrition still in its infancy, methodological issues, such as weak design, limited use of validated measures and lack of underpinning theory, have been emphasised (McGowan et al. 2017; Reicks et al. 2018). As the potential benefits of cooking are increasingly recognised, it is essential to understand whether these issues continue to persist as the research enters new target populations such as athletes.

### 1.2 Importance of Nutritional Intake for Athletes

A high‐quality diet is fundamental in sport in terms of facilitating the health, competition performance and training goals of the athlete (Thomas et al. 2016; Burke et al. 2019b, 2011; Rodriguez et al. 2009; Phillips and Van Loon 2011; Beck et al. 2015). Despite this understanding, the diet of many athletes is consistently deemed inadequate to support training and competition (Jenner et al. 2018; Tooley et al. 2015). While a systematic review of 21 studies showed that most professional sports teams meet or exceed nutritional recommendations for dietary protein and fat intake, athletes consistently failed to meet dietary recommendations for energy and carbohydrate intake (Jenner et al. 2019). Deficiencies in carbohydrate intake and relative energy deficiency in sport (RED‐S) can cause several physiological and health consequences for athletes, including reduced rates of muscle protein synthesis, impaired immune function, decreased bone mass and functional hypothalamic amenorrhea, which in turn can detrimentally impact training adaptation and performance (Dipla et al. 2021; Viner et al. 2015; Howarth et al. 2010; Oxfeldt et al. 2023). Therefore, it is essential to understand why athletes' nutritional intake is suboptimal and explore methods for enhancing dietary intake to facilitate improved performance and health.

Numerous psychosocial, socioeconomic and individual factors, such as food preferences, attitudes, motivation, mood, education, income, skill and nutrition knowledge, all influence food behaviours and nutritional intake (Köster 2009; Shepherd and Raats 2006; Birkenhead and Slater 2015). Although considerable emphasis is placed on the importance of nutrition knowledge in athlete populations, a recent systematic review concluded that nutrition knowledge is poor in athletes (Janiczak et al. 2022). Furthermore, limited consideration has been dedicated to elements of procedural knowledge, such as dietary planning and preparation, which are required to implement nutritional recommendations. Practical guidance exists regarding recommended quantities of macronutrient and micronutrient intake that are tailored to different athlete goals, such as training adaptation, improved body composition and enhanced recovery (Tipton and Witard 2007; Heaton et al. 2017; Witard et al. 2019), alongside broader nutritional strategies such as nutrient timing and dietary periodisation (Burke et al. 2019a). However, the translation of sports nutrition science into practice remains a significant challenge for athletes, coaches and applied practitioners due to a distinct lack of clear and meaningful culinary guidance. It is possible to bypass the need for self‐culinary preparation through professional services that provide meals for athletes (chef services) or the purchase and consumption of pre‐prepared meals. However, the limitations to these strategies include sporting level, that is, a minority of high‐level elite/professional athletes have access to chefs and the prohibitory cost associated with purchasing pre‐prepared meals that are tailored to athlete needs. Therefore, empowering athletes with cooking and food skills to enable self‐preparation to practically implement sports nutrition guidance is crucial for sustaining athlete health and performance.

### 1.3 Athletes and Culinary Capacity

Athletes are a unique population subgroup that, depending on their sport, competitive level, age and multiple other factors, require individual and personalised nutritional strategies tailored to the physiological demand of each sport. The responsibility of dietary intake lies with the individual athlete, coaches and wider support networks (including chefs in some situations), parents, nutritionist, dietitian or teacher. This reliance depends on the athlete's age, demographic and social characteristics. To facilitate athlete autonomy, the primary focus of culinary nutrition should be on the individual's capacity to optimise dietary quality and intake that may in turn have an initial positive influence on sports performance and could have potential longer‐term health implications for when the athlete retires. However, there is limited evidence to suggest that athletes exhibit adequate culinary skills (Jeukendrup and Gleeson 2019; Tam et al. 2019). With cooking skills and nutrition knowledge serving as two factors that could influence the adequacy of an athlete's diet and subsequent athletic performance, it is prudent to suggest that all athletes should possess cooking skills, feel comfortable in a kitchen environment and understand how to use basic kitchen appliances and utensils to build nutritious and appetising meals.

Moving forward, more robust systematic research is required to develop a better understanding of athletes' culinary skills as well as their understanding of preparation behaviours, relationship with food and barriers and facilitators to implementing culinary skills. Furthermore, while several reputable nutritional guidelines inform athletes with evidence‐based recommendations on nutritional intake (Thomas et al. 2016; International Olympic Committee 2008), it is essential to understand how effectively this science is translated into real‐world practical advice in the form of culinary nutrition interventions for athletes. Therefore, the primary aim of this systematic scoping review is to collate, map and provide an overview of the current body of literature on culinary nutrition in athletes. The secondary aim is to identify existing gaps in knowledge and provide recommendations for future research regarding culinary nutrition in athlete populations.

## 2 Materials and Methods

### 2.1 Protocol and Registration

The Preferred Reporting Items for Scoping Reviews (PRISMA‐ScR) (Appendix S1) and JBI methodology guided this review (Tricco et al. 2018). A protocol was prepared in advance and registered on the Open Science Framework (OSF) (doi.org/10.17605/OSF.IO/758GM).

### 2.2 Search Strategy

Five electronic databases were searched and included Medline (OVID), Embase (OVID), Web of Science (Web of Science), ERIC and PsycInfo (OVID). The search was conducted using keywords, MeSH and other index terms, as well as combinations of these terms and appropriate synonyms; it was developed by the first author and reviewed by the team. Search terms included, but were not limited to, culinary nutrition, cooking skills, cook*, nutrition AND athlete*, runner, cyclist, gymnast, soccer, football, rugby, swimmer. Full details regarding the complete search strategy are displayed in Appendix S2. The searches were conducted in March 2023 and were limited to original research articles that recruited human subjects and were published in the English language. No time limits on the publications were implemented to maximise the breadth of literature identified. To ensure a thorough search and identification of all relevant studies, reference lists of included studies were back‐searched and forward citation tracking was conducted using Google Scholar.

### 2.3 Study Screening and Selection

All identified citations were uploaded to Endnote (Clarivate Analytics, USA) to remove duplications. Citations were then exported to Covidence (Covidence systematic review software; Veritas Health Innovation, Melbourne, Australia) for manual screening. All titles and abstracts of identified citations were manually reviewed against the eligibility criteria by two independent reviewers (RC & MR) and FL resolved any disagreements. Full‐text articles were screened independently by two reviewers (RC & MR) with the primary reason for exclusion recorded. Any full‐text screening discrepancies were resolved by a third reviewer (FL).

### 2.4 Study Eligibility

Peer‐reviewed primary research articles were included in this review. Reports, theses, reviews and study protocols with no published results were excluded.

#### 2.4.1 Participants

Eligible studies included an athlete population of any sport/field, age and sex. Additionally, studies that involved coaches, parents, support staff, nutritionists or dietitians aimed at influencing athletes' nutritional intake were included. Studies were excluded if they were not focused on athlete populations.

#### 2.4.2 Concept

All primary peer‐reviewed published research that contained elements of culinary nutrition, cooking or culinary skills, using any study design such as cross‐sectional surveys, interviews, focus group discussions, interventions and workshops were included in the scoping review. Primary research that was not peer‐reviewed, reviews of primary research or research not providing any detail on the culinary nutrition/cooking aspect of the research was not eligible for inclusion.

#### 2.4.3 Context

The context for this review was international and in any physical setting, i.e., school, university, in training facilities and published in any year.

### 2.5 Data Extraction, Charting and Critical Appraisal of Individual Studies

A custom form was developed to facilitate relevant data extraction. Data were independently extracted initially by one reviewer (RC) and checked for accuracy (FL, OW). Data extracted included the participant characteristics (athletic profession and age), sample size, study design and aims, measured outcomes, key findings, study duration, intervention type, use of an underlying theoretical framework or model, details of the professional that developed and delivered the intervention and duration of intervention. Although not commonplace in scoping reviews, a critical appraisal was conducted due to previous reviews indicating that culinary interventions are poorly designed (Reicks et al. 2018; Taylor et al. 2021; Lavelle 2022). A critical appraisal of the current culinary research in this target population (athletes) provided initial insights as to whether methodological issues are similarly occurring in this population. Two reviewers (RC, FL) independently conducted a critical appraisal of the eligible studies using The Academy of Nutrition and Dietetics Quality Criteria Checklist for Primary Research in human subjects (Academy of Nutrition and Dietetics 2016). This tool was chosen as multiple study designs can be assessed using a single tool to assess the quality of culinary interventions in various populations (Reicks et al. 2018, Taylor et al. 2021, Lavelle 2022). Based on the responses to 10 overall validity questions, a quality rating is assigned to each eligible study and a positive, neutral or negative outcome was recorded (Dietetics, 2016). Full details of the quality assessment are displayed in Appendix S3.

### 2.6 Data Synthesis

Details regarding study design, setting, type of athlete and the culinary context were charted in narrative and numerical format in this scoping review. Thematic analysis was conducted (RC) to map relationships between studies and identify gaps. Data immersion was achieved through screening, reading and extraction phases. Included study findings were coded, and following this, codes were grouped to generate initial themes. Finally, the themes were inspected for overlap, and where necessary, refined through critical discussion (RC, FL).

## 3 Results

**FIGURE 1:**
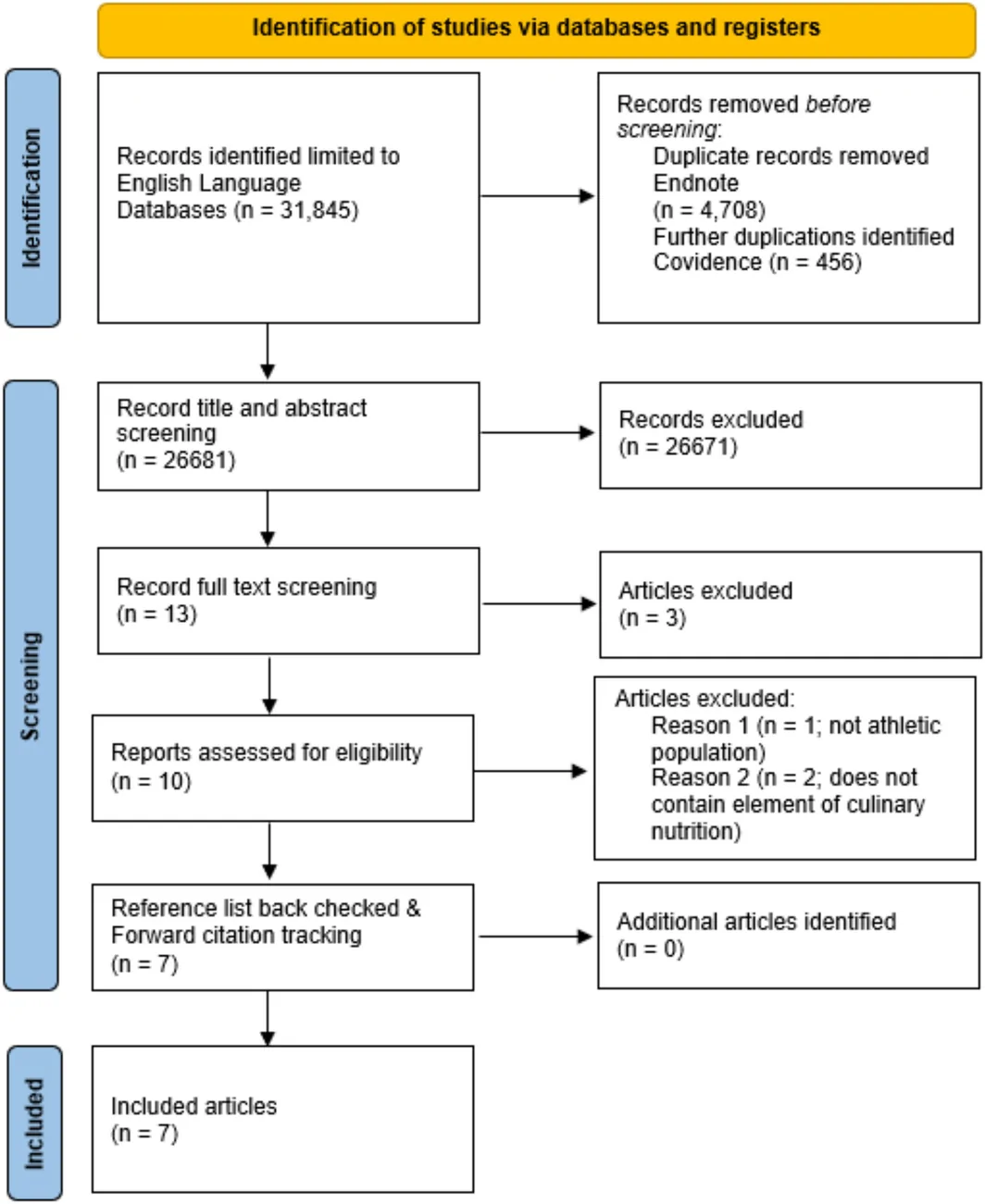
Flow diagram for screening process.

A total of 31 845 records were identified, 4708 duplicates were removed via Endnote processing, and 456 via Covidence automation. After duplication removal, a total of 26 681 articles were screened for eligibility by two reviewers. The reasons for exclusion were as follows: (1) participants were not from an athlete population and the article did not present a focus on culinary nutrition, (2) the concept of culinary nutrition or cooking was not reported and (3) the context was not relevant to culinary nutrition or cooking. Seven studies met the inclusion criteria and were included in the scoping review. The screening process is outlined in Figure 1 using Preferred Reporting Items for Systematic Reviews and Meta‐analysis for Scoping Reviews (PRISMA‐ScR) (Figure 1).

### 3.1 Study Design

Of the seven primary research articles included (Table 1) in this scoping review (Heaney et al. 2008; Ellis et al. 2018; Patton‐Lopez et al. 2018; Larose et al. 2022; Carter et al. 2022; Lydon et al. 2023; Brauman et al. 2023), five were quantitative study designs (Ellis et al. 2018; Patton‐Lopez et al. 2018; Larose et al. 2022; Lydon et al. 2023; Brauman et al. 2023). These articles included a non‐randomised controlled trial (Patton‐Lopez et al. 2018) (*n* = 217) that assessed the impact of a sports nutrition education and life skills intervention on sports nutrition knowledge, attitudes/beliefs and behaviours among high school soccer players; a randomised controlled trial (Larose et al. 2022) (*n* = 23) that assessed the impact of a nutrition intervention combining nutrition education and a cooking workshop on nutrition knowledge, intention, perceived behavioural control in preparing healthy meals, dietary intake and diet quality; and two single‐group intervention pre–post study designs (Ellis et al. 2018; Lydon et al. 2023) (*n* = 336; *n* = 22) that assessed nutrition knowledge, food preparation skills, cooking skills, attitudes towards nutrition and a teaching intervention to increase self‐efficacy for making healthy food choices. Moreover, one study was a cross‐sectional survey (Brauman et al. 2023) (*n* = 169) that identified perceived barriers to healthy eating. In addition, two qualitative studies that investigated the barriers affecting athletes' ability to consume a diet that is consistent with sports nutrition recommendations were included in this review (Heaney et al. 2008; Carter et al. 2022), one using interviews (Carter et al. 2022) (*n* = 35) and one using focus groups (Heaney et al. 2008) (*n* = 46).

**TABLE 1:** Data extraction for included studies showing study design, aims and characteristics.

Study	Study design	Study aims	Sample size (*N*)	Sex distribution	Age (years)	Participants	Athlete representation
Heaney et al. (2008)	Focus group design	To identify barriers affecting the ability of athletes to consume a diet consistent with sports nutrition recommendations	46	Male (*n* = 16; 35%) Female (*n* = 30; 65%)	16–45	Athletes, Elite coaches, sports dietitians	Diving, netball, basketball, lawn bowls, swimming, soccer, sailing, cycling, and golf
Ellis et al. (2018)	Intervention, single group pre–post‐test	To teach athletes to cook and increase their self‐efficacy for making healthful food choices and preparing food	22	Male (*n* = 13; 59%) Female (*n* = 9; 41%)	18+	Athletes	Football, men's baseball, netball, volleyball, soccer, swim/dive, and tennis
Patton‐Lopez et al. (2018)	Non‐randomised comparative study	To examine the impact of a sports nutrition education and life skills intervention on sports nutrition knowledge, attitudes/beliefs and behaviours among high school soccer players to determine differences	217	Male (*n* = 187; 86%) Female (*n* = 30; 14%)	14–19	Athletes	Soccer
Larose et al. (2022)	Randomised control trial	To assess the impact of a nutrition intervention combining nutrition education and cooking workshop on nutrition knowledge, intention and perceived behavioural control in the preparation of healthy meals, dietary intake and diet quality	23	Male (*n* = 23; 100%)	18–25	Athletes	Football (American style)
Carter et al. (2022)	Qualitative‐semi‐structured interview design	To investigate players, sports nutritionist and coaches' perspectives of the barriers and enablers to adhering to nutritional recommendations	35	Male (*n* = 32; 91%) Female (*n* = 3; 9%)	18+	Athletes, Sports Nutritionists, Coaches	Football (soccer)
Lydon et al. (2023)	Intervention—single group pre–post test	To assess nutrition knowledge, food preparation skills, cooking skills confidence, attitudes towards nutrition, future diet and food practice	336	Male (*n* = 247; 74%) Female (*n* = 89; 26%)	16+	Athletes	Gaelic athletic players (Gaelic football, hurling, handball)
Brauman et al. (2023)	Cross‐sectional survey	To identify perceived barriers to a healthy dietary intake and to identify the most significant of these barriers	169	Male (*n* = 48; 28%) Female (*n* = 121; 72%)	18+	Athletes	Baseball, softball, men's basketball, women's basketball, men's cross country, women's cross country, football, men's golf, women's golf, men's tennis, women's tennis, men's track and field, gymnastics, soccer, swimming/dive, and volleyball

### 3.2 Study Participants and Location

An overview of the study characteristics is displayed in Table 1. Of the seven studies, four included athletes from multiple sports, for example, diving, netball, basketball, lawn bowls, swimming, soccer, sailing, cycling, volleyball and tennis, Gaelic athletic players encompassing Gaelic football, hurling and court handball (Heaney et al. 2008; Ellis et al. 2018; Lydon et al. 2023; Brauman et al. 2023). Other studies included athletes from single‐origin sports, including football (soccer) (Patton‐Lopez et al. 2018; Larose et al. 2022; Carter et al. 2022). The age of athletes varied with the studies reporting a range including 18+ years (Ellis et al. 2018; Carter et al. 2022; Brauman et al. 2023), 14–19 years (Patton‐Lopez et al. 2018), 18–25 years (Larose et al. 2022) and a mean age of 16.1 years (Lydon et al. 2023). The distribution of sex across studies presented as male‐dominant, with only two non‐intervention studies having a majority of female participants (Heaney et al. 2008; Brauman et al. 2023) and the five other studies ranging from 59% (Ellis et al. 2018) to 100% (Larose et al. 2022) male participants. One study was conducted in Australia (Heaney et al. 2008), three in the USA (Ellis et al. 2018; Patton‐Lopez et al. 2018; Brauman et al. 2023), one in Canada (Larose et al. 2022), one in the UK (Carter et al. 2022) and one on the Island of Ireland (IOI) (Lydon et al. 2023).

### 3.3 Study Description

All studies were conducted in different settings (Table 2), while two did not specify the setting (Heaney et al. 2008; Ellis et al. 2018). Three studies were conducted in a classroom, during school and a school soccer camp (Patton‐Lopez et al. 2018), university campus (Larose et al. 2022) and in a school setting (Lydon et al. 2023). Two studies were conducted online (Carter et al. 2022, Brauman et al. 2023). The duration of the intervention ranged from 3‐h food skills and education workshops delivered by a home economics teacher (Lydon et al. 2023) to a two‐year classroom‐based education intervention delivered by a dietitian nutritionist (Patton‐Lopez et al. 2018). Two studies included a 4‐week intervention duration (Ellis et al. 2018; Larose et al. 2022), whereby workshops and sessions were distributed weekly and delivered by either a dietitian (Ellis et al. 2018) or a kinesiology student trained in nutrition (Larose et al. 2022).

**TABLE 2:** Population, context, concept and quality assessment of included studies.

Study	Country	Setting	Intervention duration	Cooking outcome	Underpinning theory/model	Intervention content	Delivery of intervention	Reported outcomes/findings	Quality of evidence
Heaney et al. (2008)	Australia	Not specified	NA	Not specified	Nil	NA	NA	Barriers that influence an athletes ability to eat an appropriate diet: time and financial constraints; lifestyle; physique; nutrition knowledge	Negative
Ellis et al. (2018)	USA	Not specified	4 weeks (Workshop duration not specified)	Primary	Social Cognitive Theory	Cooking education	Not specified	**Self‐efficacy for food selection** **Self‐efficacy for food preparation**	Negative
Patton‐Lopez et al. (2018)	USA	Classroom education, during school and soccer camp	2 years	Nil	Nil	Nutrition and life skills intervention	Dietitian nutritionist	**Sports Nutrition Knowledge** **Attitudes and Beliefs related to Sport Nutrition** Dietary behaviours related to Sport performance	Negative
Larose et al. (2022)	Canada	University Campus	4 weeks	Primary	Theory of Planned Behaviour	Nutrition education and cooking workshop	Kinesiology student trained in nutrition and dietetic technician	**Sports Nutrition Knowledge** Intention to prepare healthy meals Perceived behavioural control to prepare healthy meals **Dietary intake** Diet quality Sleep quality Stress levels Mood disturbances	Positive
Carter et al. (2022)	UK	Online	NA	Not specified	COM‐B Theory Domains Framework	NA	NA	Barriers and enablers to nutritional adherence: Nutritional Knowledge; Cooking Skills; Training Venue Food Provision; Nutritionist Accessibility and Approachability; Living Status; Performance Implications; Role Modelling	Neutral
Lydon et al. (2023)	Island of Ireland (IOI)	School setting	3 h	Primary	Nil	Food skills and nutrition education workshop	Home Economics Teacher	**Cooking Confidence** **Nutrition knowledge**	Negative
Brauman et al. (2023)	USA	Online	NA	Secondary	Nil	NA	NA	Barrier to consuming a healthy diet: ‘Lack of knowledge and skills to cook healthy foods’ rated fifth **Higher reported dietary intake less likely to feel they lacked knowledge and skills to cook healthy foods**	Negative

*Note:* Constructs in bold indicate a significant finding (*p* < 0.05) reported as a result of the intervention.

### 3.4 Programme Curriculum

Of the four intervention/workshop studies, one study focused on cooking education (Ellis et al. 2018), whereas three focused more broadly on nutrition education with some cooking and/or food skills (Patton‐Lopez et al. 2018; Larose et al. 2022; Lydon et al. 2023). All studies provided nutrition education tailored to a sport context and included practical hands‐on cooking session(s), with one providing gardening elements (Patton‐Lopez et al. 2018), and three specifically stating they provided the recipes to the participants (Patton‐Lopez et al. 2018, Larose et al. 2022, Lydon et al. 2023). Only one study detailed the actual content of the cooking/nutrition intervention that included information regarding the specific nutrition aspects that were targeted in each session and what recipes were included (Larose et al. 2022). With a view to developing curriculum in accordance with sports nutrition recommendations, Larose et al. used the American College of Sports Medicine (ACSM) guidelines (Larose et al. 2022), whereas Ellis and colleagues used a performance plate (Ellis et al. 2018). This performance plate appeared to be a non‐validated tool developed by sports dietitians for the intervention (Ellis et al. 2018). Similarly, Lydon and colleagues based their recipes on nutritional guidelines for optimal performance; however, they did not state the origin of the guidelines (Lydon et al. 2023).

### 3.5 Reported Outcomes and Measurement Tools

For the intervention studies, two studies reported cooking/preparation confidence or self‐efficacy (Ellis et al. 2018; Lydon et al. 2023), and preparation skills (Lydon et al. 2023), with one study using validated measures (Ellis et al. 2018) and one study using unvalidated measures (Lydon et al. 2023). One intervention study assessed intention and perceived behavioural control towards healthy meal preparation that followed the Theory of Planned Behaviour guidelines (Larose et al. 2022), with one study including no preparation related outcome measurement (Patton‐Lopez et al. 2018). Three studies reported nutrition knowledge outcomes (Patton‐Lopez et al. 2018, Larose et al. 2022, Lydon et al. 2023), with two being sport‐specific nutrition knowledge (Patton‐Lopez et al. 2018; Larose et al. 2022). One study reported using a previously validated nutrition knowledge measure although it was not previously validated (sections in the cited questionnaire were taken from previous validated measures, however, the nutrition knowledge questions were not) (Patton‐Lopez et al. 2018). Two studies reported dietary behaviours/intake and performance related outcomes (Patton‐Lopez et al. 2018; Larose et al. 2022), with one using validated dietary measures (Larose et al. 2022). The cross‐sectional study reported a ‘Lack of knowledge and skills to cook healthy foods’ as the fifth highest ranked barrier to consuming a healthy diet (Brauman et al. 2023). The survey was researcher‐developed and not validated (Brauman et al. 2023). Both qualitative studies identified nutrition knowledge, cooking skills and capability as key barriers to healthy eating in athletes (Heaney et al. 2008; Carter et al. 2022). One qualitative study reported deriving their topic guide from the literature (Heaney et al. 2008) and the other based their questions on theory and also piloted their topic guide (Carter et al. 2022).

### 3.6 Critical Appraisal

The critical appraisal and assessment of included studies (Table 2) resulted in five of the seven studies with a negative rating (Heaney et al. 2008; Ellis et al. 2018; Patton‐Lopez et al. 2018; Lydon et al. 2023; Brauman et al. 2023), one neutral (Carter et al. 2022) and one received a positive rating (Larose et al. 2022). Details of full scoring are displayed in Appendix S3.

### 3.7 Themes and Gaps

Four interrelated themes were identified among the included studies: (1) The Role of Nutrition Knowledge, (2) The Relationship between Dietary Intake and Performance, (3) Cooking for Skill Development, Independence and Healthy Eating and (4) Psychological and Theoretical Underpinnings of Behaviour. A description of themes and gaps in evidence is described in the following section.

#### 3.7.1 The Role of Nutrition Knowledge

The nutrition education and cooking workshops significantly improved nutrition knowledge (Patton‐Lopez et al. 2018; Lydon et al. 2023). Two studies reported a 10% increase in nutrition knowledge immediately after the intervention (Patton‐Lopez et al. 2018, Larose et al. 2022) that continued after a 2‐month follow‐up in one study (Larose et al. 2022). Studies also investigated knowledge as a barrier and how a lack of knowledge impacts athletes' ability to select and cook healthy foods (Brauman et al. 2023). This observation is consistent with focus group findings from Heaney and colleagues (Heaney et al. 2008), whereby coaches established a link between nutrition knowledge and appropriate nutritional behaviour. In this regard, one study reported that nutrition knowledge increased alongside reported knowledge regarding pre/post training, match‐day and performance sport nutrition recommendations in a large (*n* = 336) cohort of youth (16 years) Gaelic athletic players (Lydon et al. 2023). However, a primary issue in assessing and comparing nutrition knowledge on a large scale across different athlete populations is the lack of validated measurements; for example, only one study used a validated sports nutrition questionnaire (Larose et al. 2022).

#### 3.7.2 The Relationship Between Dietary Intake and Performance

A primary aim of any culinary or cooking intervention for athletes is to optimise athletic performance through improvements in dietary intake. Two studies reported non‐significant changes in performance outcomes after cooking interventions (Patton‐Lopez et al. 2018, Larose et al. 2022). However, by the end of one culinary nutrition intervention, players in the intervention group were three times more likely to eat for performance (Patton‐Lopez et al. 2018). Furthermore, in one intervention study, 60% of participants reported having previously sought nutrition advice to improve performance (Lydon et al. 2023), highlighting that athletes associated performance with nutrition. Players discussed this association in a qualitative study, citing how they believed a good diet positively influenced and is essential to their performance (Carter et al. 2022). Nutritionists and coaches found that linking nutrition to performance using a data‐driven approach generated interest in players (Carter et al. 2022). The limitation of measuring performance in these studies is the duration of the intervention and lack of long‐term follow‐up, as well as the lack of validated and specific measurement tools required to assess performance outcomes in athletes.

#### 3.7.3 Cooking for Skill Development, Independence and Healthy Eating

All seven studies included an element of cooking, such as within the intervention content or as a factor relating to healthy eating in the qualitative research. Players experienced increased control, independence and ownership if they possessed adequate cooking skills (Carter et al. 2022). In the same study, nutritionists and coaches highlighted the importance of appropriate cooking skills, mainly when food provision is limited, such as when the sporting organisation/club does not provide food (Carter et al. 2022). This viewpoint, of the importance of cooking skills, was more apparent in participants that resided on a university campus or lived at home with their parents. Accordingly, (Brauman et al. 2023) reported associations between a lack of knowledge and skills to cook healthy foods and lower dietary rating (how participants rated their dietary intake, [*p* = 0.009]). After a 4‐week cooking intervention (Ellis et al. 2018), all athletes reported feeling more comfortable when cooking and were interested in the cooking topics covered. Moreover, athletes recruited to this study wanted to learn how to cook more foods and highlighted the value and need for a curriculum to teach athletes cooking skills. A food skills and nutrition education workshop was found to be a significant factor in improving culinary knowledge, skills and confidence (*p* < 0.05) and using cooking appliances to prepare meals and snacks (*p* = 0.003) (Lydon et al. 2023). However, while the study by (Larose et al. 2022) included the most detail, all intervention studies lacked precise methods to describe the cooking intervention, meaning that the intervention cannot be tested, replicated and validated across athlete populations.

#### 3.7.4 Psychological and Theoretical Underpinnings of Behaviour

When developing or changing any behaviour, there are multiple factors including psychological components such as attitudes, motivations, self‐efficacy, subjective norms and perceived behavioural control that should be considered in the context of behaviour. However, only three studies included underpinning theories or models (Ellis et al. 2018; Larose et al. 2022; Carter et al. 2022), making it difficult to understand how these factors influenced culinary behaviours in athletes. The 4‐week cooking and education intervention used a cognitive psychological model, specifically the Theory of Planned Behaviour (TPB), that is often utilised in behaviour change interventions (Larose et al. 2022). However, while the TPB guidelines for designing the items in the survey were followed, the authors only assessed the intention and behavioural control components and did not assess subjective norms or attitudes (Larose et al. 2022), making it difficult to understand culinary behaviours in line with this model (Ellis et al. 2018) based their culinary intervention on the developmental model of Social Cognitive Theory which is focused on learning and increasing self‐efficacy through mastery experiences and modelling. The intervention successfully increased self‐efficacy in food selection and preparation (Ellis et al. 2018). Finally, the COM‐B model of behaviour change underpinned the design of a topic guide to understand adherence to nutrition recommendations (Carter et al. 2022). A player's ability to cook was suggested as an essential component of capability to adhere to food and nutrition guidance (Carter et al. 2022). However, players lacked motivation to cook which may then influence dietary behaviours and adherence to nutrition recommendations. Taken together, these studies highlight that initial psychological underpinnings of culinary behaviour in this group are currently lacking.

## 4 Discussion

To our knowledge, this is the first scoping review with a focus on culinary nutrition in athletes that summarises the study characteristics, athlete populations, data collection methods and outcomes. It is essential to understand culinary nutrition in athletes to consider best practice for optimising their dietary intake and in turn, their health, performance and training adaptation. Furthermore, athletes are considered role models (Reid 2017) and thus, with an influence on the general population, could be used to model healthy cooking behaviours. This scoping review aimed to provide an overview of the existing literature and highlights the gaps and paucity of research in this area. A total of seven studies were deemed eligible for inclusion, consisting of two qualitative (Heaney et al. 2008; Carter et al. 2022) and five quantitative studies (Ellis et al. 2018; Patton‐Lopez et al. 2018; Larose et al. 2022; Lydon et al. 2023; Brauman et al. 2023). Overall, the evidence is limited, heterogeneous and of poor methodological rigour. Four studies included athletes from multiple sports (Heaney et al. 2008; Ellis et al. 2018; Lydon et al. 2023; Brauman et al. 2023), with two studies focussed on football (soccer) (Patton‐Lopez et al. 2018; Carter et al. 2022) and one on American football (Larose et al. 2022). The two qualitative studies included athletes, as well as coaches and nutritionists/dietitians (Heaney et al. 2008, Carter et al. 2022). For the intervention studies, delivery, content and duration of the interventions varied, with limited detail provided in most studies. Most eligible studies focused on the importance of nutrition knowledge for athletes. Hence, the overall scientific evidence base related to culinary nutrition in athlete populations appears to be somewhat limited, heterogeneous and of poor methodological rigour. Therefore, there is limited understanding of culinary capacity within this population and how this could be used to improve their diet quality and, in turn, enhance their health and performance.

The eligible studies were conducted across multiple countries and included participants of both sexes and from a range of ages. The majority of studies were from North America (Ellis et al. 2018; Patton‐Lopez et al. 2018; Larose et al. 2022; Brauman et al. 2023), followed by Europe (Carter et al. 2022; Lydon et al. 2023) and then Australia (Heaney et al. 2008). This demographic reflects the trend of where culinary nutrition research is conducted in the general population (Reicks et al. 2018). However, in contrast to the wider culinary nutrition research field (Reicks et al. 2018), the participants were predominantly male in this review, with five studies recruiting a male majority. The two studies with greater female participation were non‐intervention studies (Heaney et al. 2008; Brauman et al. 2023). This observation is interesting as traditionally cooking has been perceived as a ‘woman's activity’ in society (Beagan et al. 2008; Mills et al. 2017b). Based on the increasing acknowledgement that cooking serves as a mechanism for optimising nutrition intake (Wolfson and Bleich 2015; Mills et al. 2017a; McGowan et al. 2016; Wolfson et al. 2020), this nutrition intervention was initially provided to male athletes and may be considered to reflect gender gaps that exist in sport and exercise research (Cowley et al. 2021; Areta and Elliott‐Sale 2022; Costello et al. 2014; Emmonds et al. 2019). However, it could also be suggested that males tend to possess more limited cooking skills in the general population (McGowan et al. 2016; Lavelle et al. 2020; Wolfson et al. 2021) and may be in greater need of intervention. Nonetheless, female athletes have consistently reported diets that are insufficient in overall energy, carbohydrate and iron intake, when compared to health and sports performance recommendations (Renard et al. 2021) and poor nutrition knowledge (Renard et al. 2020). Additionally, a recent study highlighted that both male and female athletes exhibit lower cooking skills confidence than general populations (Renard et al. 2023) and therefore culinary nutrition interventions should be provided to all athletes. Consistent with providing culinary nutrition education to all athletes, this provision should be considered across all age groups. The studies in this review included a range of ages from 14 years old (Patton‐Lopez et al. 2018), which is encouraging as learning cooking skills at younger ages has been shown to exhibit a positive influence on diet quality and the skills are maintained into adulthood (Lavelle et al. 2016b; Laska et al. 2012; Utter et al. 2018). Therefore, it appears logical to teach athletes cooking skills at earlier ages to ensure optimal food and nutrient intake throughout their career as well as post career.

The findings of this scoping review indicate a lack of transparency in curriculum content, a lack of long‐term follow‐up and inconsistency in the delivery and durations of interventions. For instance, Larose et al. (2022) were the only study to use credible sport nutrition guidelines (American College of Sports Medicine, American Dietetic Association, Dietitians of Canada 2000) to direct the cooking workshop content specific to macronutrient, micronutrient and fluid recommendations in American football players. In comparison, other studies utilised unspecified recommendations or a performance plate developed for the intervention (Ellis et al. 2018; Lydon et al. 2023). A plate approach may be limited in this population, as plates are based on ratios and macronutrients are periodised to reflect demand, meaning there could be daily variations. The duration of interventions varied in the present review from 3 h (Lydon et al. 2023) to 2 years (Patton‐Lopez et al. 2018). It should be noted that this 2‐year intervention duration could have been a limiting factor in outcome quality, due to only a 51% retention rate over the 2 years (Patton‐Lopez et al. 2018). A lack of detail in relation to intervention description in several studies made it difficult to determine the duration and frequency of face‐to‐face contact. In this regard, the duration and frequency of an intervention are crucial to understanding the effectiveness of an intervention for improving cooking skills in an athlete population. Accordingly, a systematic review by Reicks et al. (2018) reported variations in the frequency and duration of culinary interventions in general populations, with a majority of interventions < 16 weeks in duration and the frequency of intervention ranging from 47 short videos to 70–90 min of face‐to‐face contact each week. Furthermore, this scoping review also highlighted the diversity in professionals delivering the interventions, including sports nutritionists and dietitians (Patton‐Lopez et al. 2018), a home economics teacher (Lydon et al. 2023) and a kinesiology student trained in nutrition (Larose et al. 2022). One study did not report who delivered the intervention (Ellis et al. 2018). Diversity in those delivering the interventions is a similar trend to culinary research in general populations (Reicks et al. 2018).

A critical appraisal was conducted as part of the present scoping review to understand the methodological quality of included research studies. This initiative was conducted as previous reviews indicate that culinary nutrition interventions generally lack methodological rigour (Reicks et al. 2018; Taylor et al. 2021; Lavelle 2022). Thus, it was imperative to assess if these issues are persistent in this target athlete population considering the recent nature of the research. Interestingly, only one of seven studies received a positive quality assessment rating (Larose et al. 2022), one neutral (Carter et al. 2022) and the remaining negative (Heaney et al. 2008; Ellis et al. 2018; Patton‐Lopez et al. 2018; Lydon et al. 2023; Brauman et al. 2023). Hence, it appears that methodological issues remain in culinary nutrition research. While both qualitative studies recruited adequate sample sizes and discussed how adequacy was assessed (Heaney et al. 2008; Carter et al. 2022), none of the quantitative studies conducted sample size calculations; hence, it is unclear whether these studies were sufficiently powered to detect the hypothesised effect (Whitley and Ball 2002). Additionally, only one randomised controlled study was identified (Larose et al. 2022). Studies lacked sufficient detail in terms of intervention description and content (Ellis et al. 2018) and failed to report key information such as participants' social and demographic characteristics (Carter et al. 2022).

Consistent with the wider culinary nutrition research (McGowan et al. 2017; Reicks et al. 2018) is the lack of use of validated measurement tools. The continued use of unvalidated measures is a cause for concern regarding the validity and true effect of interventions across the culinary nutrition research community. Only one study used a validated cooking self‐efficacy measure (Ellis et al. 2018), and one study used a validated web‐based 24‐h dietary recall (Larose et al. 2022). Moreover, one study erroneously reported using a previously validated nutrition knowledge measure (Patton‐Lopez et al. 2018) that had been developed for a previous study (Walsh et al. 2011); however, it was reported to be based on previous literature, but no psychometric testing was reported to assess its validity (Walsh et al. 2011). Additionally, Larose et al. (2022) acknowledged the limitation that their nutrition knowledge measure was researcher developed and only reported internal consistency reliability. Questionnaires must undergo rigorous psychometric testing to determine their validity, reliability and temporal stability to assess how effectively a questionnaire measures the concept it was designed to measure and that they are consistent over time. While validated nutrition questionnaires for athletes have been developed (Edmonds et al. 2023; Tam et al. 2021, 2020), the adapted version of PEAKS‐NQ to UK/Ireland showed unacceptable internal reliability for the subsections and only assessed content validity, neglecting temporal stability. Furthermore, several validated cooking‐related measures have recently been developed (Lahne et al. 2017; Karlsson et al. 2023; Lavelle et al. 2017; Goni et al. 2022; Dean et al. 2021a) that could be used to assess culinary nutrition interventions in this population. Methodological issues such as the poor validity of assessment tools used in culinary interventions should be addressed in future culinary research in athlete populations.

This review charted the outcomes of interest in culinary nutrition programmes within this athlete population. Although all interventions included a culinary element, one study did not report a cooking/food preparation outcome (Patton‐Lopez et al. 2018). The two studies that investigated self‐efficacy/confidence in cooking (Ellis et al. 2018, Lydon et al. 2023) reported increases in self‐efficacy/confidence post intervention, which is important as it contributes to an individual's perceived competence (the perception an individual has concerning their abilities) and is a key indicator for performing a behaviour (Harter 1978). Despite the goal of improving athletes' dietary intake, only one study measured this (Larose et al. 2022); however, this was not validated against energy expenditure data or Goldberg cut‐offs for energy intake (Black 2000). The primary target for improvement within these interventions appeared to be nutrition knowledge. The findings in this review indicate that athletes lack nutrition knowledge, with two studies reporting a 10% increase in nutrition knowledge following a cooking and nutrition education intervention (Patton‐Lopez et al. 2018; Larose et al. 2022). This observation is consistent with literature suggesting that athletes of all ages lack nutritional knowledge and are not meeting nutrient requirements (Janiczak et al. 2022; Bird and Rushton 2020; Riviere et al. 2021). This review also emphasises that cooking interventions with an education element could be used as a tool to improve nutrition knowledge in athletes. Cooking skills have been previously shown to be able to influence individuals' diet quality (Wolfson and Bleich 2015; Mills et al. 2017a). However, future studies are warranted to assess dietary intake in this population, to understand the true effect of cooking interventions on an athlete's diet. While adopting a recommended diet confers clear potential to positively impact performance outcomes, other factors aside from nutritional factors can affect performance (Burns et al. 2022) and can be grouped as environmental, psychosocial, feedback regulation, equipment and distraction (Beck et al. 2015; Currell 2014) which also require consideration when interpreting culinary intervention results. In addition, culinary interventions are typically of short duration, and although diet quality and intake may change depending on the duration of the intervention, these factors may not change sufficiently to impact an immediate measurable performance outcome. Hence, sport performance may not necessarily be the most appropriate means to measure the success of culinary interventions.

The findings from the non‐intervention studies within this review provide an indication of why nutrition knowledge, cooking and performance are of interest within this population. The qualitative studies (Heaney et al. 2008, Carter et al. 2022) among athletes and their support networks (i.e., parents, coaches and sports nutrition practitioners) highlighted that nutrition knowledge, cooking and performance are conceptually interrelated. Furthermore, in addition to the survey study (Brauman et al. 2023), these studies detail how these concepts interact as barriers and enablers to consuming a healthy diet and following food and nutrition recommendations. These concepts and socioeconomic factors should be considered in the developmental stages of culinary interventions to ensure positive outcomes. Moreover, carefully conducted qualitative studies are also essential in culinary research to understand the needs of athlete populations. Defining the needs of athletes early is a key consideration in planning a cooking intervention, as indicated by the Cook‐Ed model (Asher et al. 2020). Cook‐Ed is the first comprehensive model developed for the planning, implementation and evaluation of cooking programmes in clinical, community, research or education settings (Asher et al. 2020).

Finally, this scoping review reflects reviews in wider culinary research that highlight the existence of limited underpinning theory utilised in this area. Three studies in this review included theoretical underpinnings (Ellis et al. 2018; Larose et al. 2022; Carter et al. 2022). Ellis and colleagues (Ellis et al. 2018) used Social Cognitive Theory, a developmental theory commonly used across cooking interventions, where theory is used (McGowan et al. 2017; Reicks et al. 2018; Lavelle 2022), whereas (Larose et al. 2022) and (Carter et al. 2022) framed their research within behaviour change models. According to recent studies, only 20% of available behaviour change theories are employed in sports nutrition interventions (Bentley et al. 2020), indicating this issue is prevalent across sports nutrition and the need for research designs that utilise theoretical underpinnings to assess their effectiveness. Theoretical frameworks facilitate a greater understanding of the psychosocial factors influencing behaviour change or development and enable examination of the mechanisms and central processes (Prestwich et al. 2014; Fleury et al. 2018), which leads to useful information beyond a pure outcomes‐focused approach (Fleury et al. 2018). Given the need for theoretical/psychological underpinnings, the content of the intervention and the target population, a multidisciplinary approach to designing interventions in this area may be beneficial. Input from sports coaches, dietitians/nutritionists, home economists/chefs and psychologists/behavioural scientists could strengthen the design of interventions in culinary nutrition.

### 4.1 Summary of Recommendations for Future Research

Due to the paucity of research in this area, there are numerous directions that culinary research could take within an athlete population. However, it is essential that the foundation of this research is solid, and further consideration is needed to ensure the strength of the design and assessment. Additional qualitative research in relation to the barriers and enablers to adhering to sports nutrition recommendations, particularly in relation to how cooking interacts with these, would provide guidance on specific areas to target. This aligns to the CookEd model (Asher et al. 2020), where initial exploration of the problem is needed. This could be further supported with qualitative research exploring the barriers and facilitators to cooking that are specific to this population and are additional to those identified in the general population such as time, cost and effort (Lavelle et al. 2016a; Wolfson et al. 2016). This initial exploratory research could identify different motivators to target cooking behaviours such as learning new skills, gaining independence or, depending on the age of the athletes, potentially changing food behaviours for sustainability reasons, since young people are more open to changing their behaviours for environmental sustainability (Piscitelli and D'uggento 2022; Pieniak et al. 2016). These interventions should be applied across all age groups, potentially for different purposes. For example, in children it may be to target fine motor movement which in turn could enhance sporting performance (Dean et al. 2021b), in adolescents, the target may be to increase confidence and independence (Lavelle et al. 2016a), whereas, in adults the purpose may be to change specific dietary components and the intervention is providing the skills to enable that change. However, this additionally highlights how the outcome being measured needs to be appropriate for the type and duration of an intervention. Similarly to obesity being questioned as a reasonable outcome measure in children's cooking interventions (Lavelle 2022), sport performance could be considered inappropriate in this population. Sport performance is a complex and multifaceted outcome and due to the relatively low dosage and short duration of culinary nutrition interventions, it seems an unattainable target to impact upon over the time frame of an intervention.

The issues in methodologies, previously highlighted, need to be considered in future research to help strengthen this area. Studies need to be thoroughly designed, and where feasible, randomised controlled trials should be conducted; although a key point for all intervention studies is to conduct follow‐up measurements. This will enable understanding of long‐term changes, as follow‐up assessments are limited in the culinary nutrition area generally. The design should be based on underpinning theory, for example psychological learning theories such as Social Learning Theory (Bandura and Walters 1977) or Experiential Learning Theory (Kolb 2014) or behaviour theories such as the COM‐B system (Michie et al. 2011). The theory should not just be vaguely referenced, but how the theory informs the design should be explicitly detailed. Additionally, a comprehensive description of the content should be included to enable understanding and repetition where appropriate. Furthermore, the content should be designed based on relevant sports nutrition recommendations for the target population. The meals/recipes included should encompass appropriate cooking skills to achieve these recommendations (Dean et al. 2021b; Asher et al. 2022a). Finally, where possible, validated measurement tools should be used or validation/reliability testing should be conducted on new tools and clearly described. In the case of qualitative research, topic guides should be based on previous research and/or designed based on theory and piloted for clarity and understanding. Strengthening assessment is vital to understand the validity and true effect of intervention studies. The continued use of unvalidated measurement tools in the area of culinary nutrition is a significant cause for concern (McGowan et al. 2017; Reicks et al. 2018; Taylor et al. 2021; Lavelle 2022).

### 4.2 Strengths and Limitations

This scoping review is the first to comprehensively examine culinary nutrition literature focused on athletes and has multiple strengths. First, the review adhered to the recommended guidelines at all stages offered by the PRISMA‐ScR checklist (Tricco et al. 2018) and two independent reviewers conducted all stages of this review. As a result, the search strategy across five databases identified over 31 000 studies. Second, the broad choice of search terms encompassed nutrition and culinary nutrition across various sporting fields; however, it is acknowledged that some search terms may be missing. Third, a critical appraisal of all included studies was conducted using a validated tool adaptable for multiple study designs (Academy of Nutrition and Dietetics 2016). However, some limitations of this review must be considered. Due to limited time and resources, several sport‐specific databases were not searched, such as SPORTDiscus. However, a thorough search strategy was conducted across five databases, and forward and back citation tracking and reviewing were used, and no further studies were identified from the forward and back citation tracking. Finally, only peer‐reviewed publications in the English language were included; hence, it is likely that other culinary nutrition programmes/interventions exist in practice that have not been published or are available in additional languages.

## 5 Conclusion

This review comprehensively describes the current body of published literature on culinary nutrition focused on athletes and identifies gaps in the existing literature. Overall, the evidence is limited, heterogenous and of negative quality. Future recommendations for culinary research include well‐designed studies that utilise a clear theoretical framework as well as the development of validated measurements to strengthen this emerging area of research. By utilising a multidisciplinary approach and strong study design, culinary interventions have the potential to positively influence nutrition knowledge, food preparation and cooking skills, dietary intake and performance across all ages and competition levels and provide athletes with autonomy around nutrition. Furthermore, in their capacity as role models for the public, it is essential to understand and enhance their culinary skills to enable athletes to model healthy culinary behaviours.

## Author Contributions

Conceptualisation (F.L. and O.W.). Developed the research protocol (R.C., F.L. and O.W.). Citation screening (R.C., M.R. and F.L.). Data extraction and checking (R.C., F.L. and O.W.). Critical Appraisal (R.C. and F.L.). Manuscript drafting (R.C., F.L., O.W., M.R. and C.O.C.). All authors read and approved the final manuscript.

## Conflicts of Interest

The authors declare no conflicts of interest.

## Supporting information


Appendix S1.

Appendix S2.

Appendix S3.

## Data Availability

The authors have nothing to report.
