# Supplementary material for: Do Athletes Cook? A Systematic Scoping Review of Culinary Nutrition in Athletes
Source: Nutr Bull. 2025 May 7;51(3):322–36. doi: 10.1111/nbu.70012 (PMC13522870; doi:10.1111/nbu.70012)
Supplement: Supplementary file 2 [file NBU-51-322-s003.docx]

**Key words**

Population search: (Athlet* OR runner OR swim* OR gymnast OR rubgy OR football* OR fighter OR “martial arts” OR olymp* OR dancer OR “endurance sport” Or sporting OR sportswomen OR sportsman OR “sport competitor” OR “professional athlete” OR weightlifting OR marathoner OR cyclist OR soccer OR triathl* OR biathl* OR ironman OR para-athlete)

Concept search: (Culinary OR “Culinary nutrition” OR ‘Culinary skills’ OR “cooking skills” OR “cooking knowledge” OR cook* OR “culinary classes” OR recipe OR nutrition OR “cooking knowledge” OR “culinary knowledge” Or “nutrition education” OR “cooking education”)

Search strategy:

**Search strategy results from databases:**

| Databases | Platform | Records retrieved after adjustment for English language |
| --- | --- | --- |
| Embase (Biomed) | OVID | 6442 |
| Medline (Medicine) | OVID | 2765 |
| Web of Science | WOS | 21,861 |
| PsycInfo (Psychology) | OVID | 434 |
| ERIC | ERIC | 340 |

**Key word search strategy for Medline (OVID)**

| **#** | **Search terms** | **Results (n)** |
| --- | --- | --- |
| **1** | Athlet* | 107733 |
| **2** | Runner | 2350 |
| **3** | Swim* | 52125 |
| **4** | Gymnast | 424 |
| **5** | Rugby | 4070 |
| **6** | football | 15834 |
| **7** | fighter | 1046 |
| **8** | Marial arts | 1 |
| **9** | Olymp* | 7578 |
| **10** | Dancer | 723 |
| **11** | Ednurance Sport | 206 |
| **12** | Sporting | 6373 |
| **13** | Sportswomen | 153 |
| **14** | Sportsman | 263 |
| **15** | Sport competition | 178 |
| **16** | Pofessional athlete | 218 |
| **17** | Weightlifting | 870 |
| **18** | Marathoner | 50 |
| **19** | Cylcist | 1207 |
| **20** | Soccer | 14987 |
| **21** | Para-athlete | 35 |
| **22** | Triathlon | 1077 |
| **23** | Triathlete | 205 |
| **24** | Biathlete | 5 |
| **25** | Biathlon | 130 |
| **26** | Ironman | 373 |
| **27** | 1 or 2 or 3 or 4 or 5 or 6 or 7 or 8 or 9 or 10 or 11 or 12 or 13 or 14 or 15 or 16 or 17 or 18 or 19 or 20 or 21 or 22 or 23 or 24 or 25 or 25 | 180650 |
| **28** | Culinary | 2568 |
| **29** | Culinary nutrition | 26 |
| **30** | Culinary skills | 52 |
| **31** | Cooking | 28286 |
| **32** | Cooking skills | 280 |
| **33** | Cook | 6231 |
| **34** | Nutrition | 274077 |
| **35** | Cooking knowledge | 13 |
| **36** | Cooking education | 29 |
| **37** | 28 or 29 or 30 or 31 or 32 or 33 or 34 or 35 or 36 | 307228 |
| **38** | 27 and 37 | 2915 |
| **39** | Limit 38 to English language | 2765 |

**Key word search strategy for Embase (OVID)**

| **#** | **Search terms** | **Results (n)** |
| --- | --- | --- |
| **1** | Athlet* | 115975 |
| **2** | Runner | 7638 |
| **3** | Swim* | 66373 |
| **4** | Gymnast | 741 |
| **5** | Rugby | 5312 |
| **6** | football | 19225 |
| **7** | fighter | 5160 |
| **8** | Marial arts | 2 |
| **9** | Olymp* | 42403 |
| **10** | Dancer | 1590 |
| **11** | Endurance Sport | 1296 |
| **12** | Sporting | 9175 |
| **13** | Sportswomen | 202 |
| **14** | Sportsman | 484 |
| **15** | Sport competition | 207 |
| **16** | Pofessional athlete | 1026 |
| **17** | Weightlifting | 1074 |
| **18** | Marathoner | 61 |
| **19** | Cylcist | 2472 |
| **20** | Soccer | 14670 |
| **21** | Para-athlete | 31 |
| **22** | Triathlon | 1927 |
| **23** | Triathlete | 492 |
| **24** | Biathlete | 8 |
| **25** | Biathlon | 143 |
| **26** | Ironman | 469 |
| **27** | 1 or 2 or 3 or 4 or 5 or 6 or 7 or 8 or 9 or 10 or 11 or 12 or 13 or 14 or 15 or 16 or 17 or 18 or 19 or 20 or 21 or 22 or 23 or 24 or 25 or 25 | 251281 |
| **28** | Culinary | 3327 |
| **29** | Culinary nutrition | 53 |
| **30** | Culinary skills | 108 |
| **31** | Cooking | 30846 |
| **32** | Cooking skills | 386 |
| **33** | Cook | 24708 |
| **34** | Nutrition | 379585 |
| **35** | Cooking knowledge | 21 |
| **36** | Cooking education | 37 |
| **37** | 28 or 29 or 30 or 31 or 32 or 33 or 34 or 35 or 36 | 432783 |
| **38** | 27 and 37 | 6828 |
| **39** | Limit 38 to English language | 6442 |

**Key word search strategy for Psychinfo**

| **#** | **Search terms** | **Results (n)** |
| --- | --- | --- |
| **1** | Athlet* | 33483 |
| **2** | Runner | 388 |
| **3** | Swim* | 12301 |
| **4** | Gymnast | 59 |
| **5** | Rugby | 911 |
| **6** | football | 5428 |
| **7** | fighter | 593 |
| **8** | Marial arts | 6 |
| **9** | Olymp* | 2359 |
| **10** | Dancer | 488 |
| **11** | Ednurance Sport | 50 |
| **12** | Sporting | 3242 |
| **13** | Sportswomen | 98 |
| **14** | Sportsman | 56 |
| **15** | Sport competition | 303 |
| **16** | Pofessional athlete | 84 |
| **17** | Weightlifting | 459 |
| **18** | Marathoner | 7 |
| **19** | Cylcist | 348 |
| **20** | Soccer | 4479 |
| **21** | Para-athlete | 9 |
| **22** | Triathlon | 164 |
| **23** | Triathlete | 33 |
| **24** | Biathlete | 1 |
| **25** | Biathlon | 23 |
| **26** | Ironman | 43 |
| **27** | 1 or 2 or 3 or 4 or 5 or 6 or 7 or 8 or 9 or 10 or 11 or 12 or 13 or 14 or 15 or 16 or 17 or 18 or 19 or 20 or 21 or 22 or 23 or 24 or 25 or 25 | 53585 |
| **28** | Culinary | 488 |
| **29** | Culinary nutrition | 10 |
| **30** | Culinary skills | 24 |
| **31** | Cooking | 2277 |
| **32** | Cooking skills | 161 |
| **33** | Cook | 3225 |
| **34** | Nutrition | 27525 |
| **35** | Cooking knowledge | 11 |
| **36** | Cooking education | 10 |
| **37** | 28 or 29 or 30 or 31 or 32 or 33 or 34 or 35 or 36 | 32699 |
| **38** | 27 and 37 | 459 |
| **39** | Limit 38 to English language | 434 |

**Key word search strategy for ERIC**

| **#** | **Search terms** | **Results (n)** |
| --- | --- | --- |
| **1** | Athlet* | 33483 |
| **2** | Runner | 388 |
| **3** | Swim* | 12301 |
| **4** | Gymnast | 59 |
| **5** | Rugby | 911 |
| **6** | football | 5428 |
| **7** | fighter | 593 |
| **8** | Marial arts | 6 |
| **9** | Olymp* | 2359 |
| **10** | Dancer | 488 |
| **11** | Ednurance Sport | 50 |
| **12** | Sporting | 3242 |
| **13** | Sportswomen | 98 |
| **14** | Sportsman | 56 |
| **15** | Sport competition | 303 |
| **16** | Pofessional athlete | 84 |
| **17** | Weightlifting | 459 |
| **18** | Marathoner | 7 |
| **19** | Cylcist | 348 |
| **20** | Soccer | 4479 |
| **21** | Para-athlete | 9 |
| **22** | Triathlon | 164 |
| **23** | Triathlete | 33 |
| **24** | Biathlete | 1 |
| **25** | Biathlon | 23 |
| **26** | Ironman | 43 |
| **27** | 1 or 2 or 3 or 4 or 5 or 6 or 7 or 8 or 9 or 10 or 11 or 12 or 13 or 14 or 15 or 16 or 17 or 18 or 19 or 20 or 21 or 22 or 23 or 24 or 25 or 25 | 53585 |
| **28** | Culinary | 488 |
| **29** | Culinary nutrition | 10 |
| **30** | Culinary skills | 24 |
| **31** | Cooking | 2277 |
| **32** | Cooking skills | 161 |
| **33** | Cook | 3225 |
| **34** | Nutrition | 27525 |
| **35** | Cooking knowledge | 11 |
| **36** | Cooking education | 10 |
| **37** | 28 or 29 or 30 or 31 or 32 or 33 or 34 or 35 or 36 | 32699 |
| **38** | 27 and 37 | 459 |
| **39** | Limit 38 to English language | 434 |

**Key words search strategy using Web of science**

| **#** | **Search terms** | **Results (n)** |
| --- | --- | --- |
| **1** | Athlet* | 120,994 |
| **2** | Runner | 24,467 |
| **3** | Swim* | 81,164 |
| **4** | Gymnast | 3,198 |
| **5** | Rugby | 8,905 |
| **6** | football | 37,850 |
| **7** | fighter | 9,071 |
| **8** | Marial arts | 4,600 |
| **9** | Olymp* | 60,380 |
| **10** | Dancer | 11,456 |
| **11** | Endurance Sport | 20,553 |
| **12** | Sporting | 205,145 |
| **13** | Sportswomen | 392 |
| **14** | Sportsman | 2,587 |
| **15** | Sport competition | 20,010 |
| **16** | Professional athlete | 9,866 |
| **17** | Weightlifting | 1,386 |
| **18** | Marathoner | 346 |
| **19** | Cylcist | 10,804 |
| **20** | Soccer | 31,287 |
| **21** | Para-athlete | 195 |
| **22** | Triathlon | 1,875 |
| **23** | Triathlete | 291 |
| **24** | Biathlete | 17 |
| **25** | Biathlon | 264 |
| **26** | Ironman | 670 |
| **27** | 1 or 2 or 3 or 4 or 5 or 6 or 7 or 8 or 9 or 10 or 11 or 12 or 13 or 14 or 15 or 16 or 17 or 18 or 19 or 20 or 21 or 22 or 23 or 24 or 25 or 25 | 359,421 |
| **28** | Culinary | 10,082 |
| **29** | Culinary nutrition | 43 |
| **30** | Culinary skills | 100 |
| **31** | Cooking | 92,444 |
| **32** | Cooking skills | 452 |
| **33** | Cook | 248,212 |
| **34** | Nutrition | 1,128,095 |
| **35** | Cooking knowledge | 28 |
| **36** | Cooking education | 40 |
| **37** | 28 or 29 or 30 or 31 or 32 or 33 or 34 or 35 or 36 | 1,366,840 |
| **38** | 27 and 37 | 1,769,155 |
| **39** | Limit 38 to English language | 1,965,261 |
